# Male Perineal Carcinoma: Experience in 4 Cases and Literature Review

**DOI:** 10.1155/2022/4466602

**Published:** 2022-08-22

**Authors:** Agustín Fraile Poblador, Manuel Hevia Palacios, Manuel Rodríguez Vegas, Alberto Artiles Medina, Enrique Sanz Mayayo, Silvia García Barreras, Guillermo Fernández Conejo, Rafael Rodríguez Patrón, Varona Crespo Constatino, Ana Saiz González, Javier Burgos Revilla

**Affiliations:** ^1^Urology Department, IRYCIS (Institute of Biosanitary Research of the Ramon y Cajal Hospital), Crta Colmenar Viejo, Km 9, 100, 28034 Madrid, Spain; ^2^Plastic Surgery Department, IRYCIS (Institute of Biosanitary Research of the Ramon y Cajal Hospital), Crta Colmenar Viejo, Km 9, 100, 28034 Madrid, Spain; ^3^Anatomic Pathology Department, IRYCIS (Institute of Biosanitary Research of the Ramon y Cajal Hospital), Crta Colmenar Viejo, Km 9, 100, 28034 Madrid, Spain

## Abstract

Perineal carcinoma of unknown origin is a rare and aggressive disease, so an early diagnosis and adequate treatment are essential to prevent its progression. We report the first series of cases of perineal carcinoma of unknown origin: (I) a 62-year-old male patient being followed up for a urethral stricture treated with periodic dilations with subsequent development of perineal abscesses and perineal carcinoma; (II) a 67-year-old male patient who consults for urinary discomfort associated with a perineal abscess. Recurrence of the abscess in the first month revealed the presence of an underlying perineal carcinoma; (III) a 78-year-old male patient that underwent urethroplasty with graft with subsequent regimen of periodical dilations. Recurrent formation of perianal abscesses revealed the presence of an underlying perineal carcinoma; and (IV) a 78-year-old male patient with history of in situ penile carcinoma treated by glans resurfacing. He consulted for penile pain, and imaging tests revealed a perineal abscess adjacent to the left corpus cavernosum. The core needle biopsy revealed a squamous cell carcinoma. Penile exploration and negative glans biopsy ruled out possible recurrence of penile carcinoma. The form of presentation of the disease has been very similar in all patients, demonstrating the presence of perineal abscess in all cases. Two patients had inguinal lymph node disease at diagnosis. All patients were treated by surgery, and three of them required adjuvant systemic treatment. Surgery combined with systemic treatment is probably the best option if the patient's conditions allow it.

## 1. Introduction

Carcinomas of unknown primary (CUP) origin represent 3-5% of all malignant tumors. Squamous cell CUP accounts for 5-10% of CUP, and the involvement of the male pelvis is extremely rare [1]. Perineal carcinoma can be derived, as in our case, from a process of inflammation and chronic infection in a patient with a urological history. Inflammatory process sustains the proliferation and survival of malignant transformed cells, can promote angiogenesis and metastatic processes, can negatively regulate the antitumoral adaptive and innate immune responses, and may alter the efficacy of therapeutic agents [2]. Initial presenting symptoms include buttock pain, rectal urgency, constipation, diarrhea, and urinary frequency [1]. The uniqueness of this series of cases is supported by the lack of published case reports. Surgical intervention remains the leading form of therapy; however, approaches are case specific, depending on the location of the tumor and the degree of invasiveness [3].

## 2. Case Presentation

### 2.1. Case 1

A 62-year-old male patient with a history of bulbar urethral stricture was treated with periodic dilations for three years. In this context, the patient develops a urethro-cutaneous fistula that was treated conservatively with a suprapubic and a bladder catheter.

A combined cystography is performed through the suprapubic catheter and the bladder catheter, which reveals an extravasation of urine is observed at the level of the bulbar urethra compatible with urethral fistula. The endoscopic examination revealed a devitalized penile and bulbar urethra without finding a fistulous orifice. Conservative treatment fails, and the patient presents perineal abscesses that require treatment up to four times, two of them with percutaneous drainage and the other two by open surgery. The microbiological examination revealed the presence of gram +, gram -, and anaerobic bacteria. The patient was treated with piepracillin/tazobactan associated with IV metronidazole. During the follow-up, three months later, the patient underwent several CT scans in which anfractuous collection located in the root of the penis was observed, with approximate measurements of 66 × 25 mm, unchanged from previous studies. There are no images to suggest the presence of tumor tissue. Three months later and after the failure of conservative management, the case is discussed in the clinical session together with the other members of the urology department, and it is decided to perform a Wallace-type urinary diversion and a Friedrich of the perineal wound. The patient presented a good evolution after the intervention, and the pathological result of the debridement of the perineal wound was a well-differentiated perineal carcinoma that widely affects the margins of the analyzed tissue. An extension study was carried out with PET-CT after administration of 18-FDG in which a voluminous heterogeneous perineal mass was observed, with a significant increase in metabolism, affecting both the corpora cavernosa and spongiosum body, and involves the left ischiopubic branch as well as the anal canal. The prostatic apex is affected by contiguity.

Bilateral inguinal lymphadenopathies of up to 11 mm are observed with increased metabolic activity. The case was presented in the multidisciplinary oncology committee, and it was decided to perform a complete pelvic exenteration with inguinal lymph node dissection and total penectomy.

**Figure 1:**
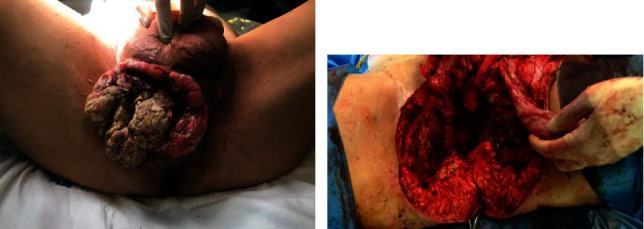
Locally advanced perineal carcinoma. (a) A voluminous and heterogeneous perineal mass. (b) Total penectomy and pelvis exenteration with resection of both ischiopubic branches.

The surgery consisted of two phases:

- Phase I: A complete pelvic exenteration with iliac-obturator lymph node dissection was done
- Phase II: Incision was made with a cold scalpel over the limits of the mass in the cutaneous plane. The bulbar urethra and the corpora cavernosa were removed from their roots. Penile degloving was performed, and the penis was extracted through the perineal incision, thus achieving an en bloc removal of all tumor tissue. Osteotomy and resection of both ischiopubic branches were performed for on block removal of surgical piece (Figure 1). Bilateral inguinal lymphadenectomy was performed

A vacuum-assisted closure system (VAC) was placed to perform a delayed closure.

Perineal closure was performed with the help of the plastic surgery department with a latissimus dorsi flap and a PTFE-polypropylene mesh. The patient remained hospitalized for 3 months. The functional recovery was excellent, allowing the patient the autonomy necessary to continue home rehabilitation.

The pathological result of specimen resulted in a well-differentiated squamous cell carcinoma of the perineal region of 11 cm in diameter that massively infiltrates the soft tissues of the perineum, spreading and infiltrating the penile root, prostatic capsule, and perianal and peri-bladder adipose tissue, affecting the bone fragments of the pubis and ischium without infiltrating the cortex. Two lymph node metastases were observed in the left inguinal lymph node dissection piece containing 7 lymph nodes in total. The patient subsequently received adjuvant treatment with four cycles of carboplatin and gemcitabine. After 3 year of follow-up, he continues with periodic reviews with PET-CT without recurrence of his disease with excellent functional recovery.

### 2.2. Case 2

A 67-year old male patient with a medical history of diabetes mellitus, chronic kidney disease, and dyslipidemia who consults for urinary discomfort, dysuria, and frequency for three months was associated with a perineal abscess. The cystography shows a contrast extravasation at the level of the bulbar urethra compatible with urethral fistula, and the endoscopic examination shows a devitalized bulbar urethra with a stenotic area that prevents the endoscope from passing. During the exploration, abundant saline solution was observed through the perineum. Surgical drainage of the collection was performed as well as placement of a bladder and suprapubic catheter. Microbiological examination did not show growth of bacteria or fungi, despite which he was treated with piperacillin/tazobactan. In the first month of follow-up, the patient returned to the emergency room due to pain at the scrotal level and at the root of the penis associated with the recurrence of the collection, so it was decided to perform a new drainage of the abscess and a Friedrich of the perineal wound.

The anatomopathological outcomes of the latter revealed the existence of a well-differentiated squamous cell carcinoma in areas of abundant keratinization that widely infiltrate the skin and soft tissue. On physical examination, the patient presents a perineal wound with drainage of purulent material, indurated on palpation, and adhered to deep planes.

CT scan and MRI reveal a mass with irregular borders with a maximum diameter of 84 mm that invades both corpora cavernosa and the corpus spongiosum, reaching the urethral bulb. The existence of metastases was excluded. After discussing the case in the multidisciplinary tumor committee, the surgical treatment was decided.

**Figure 2:**
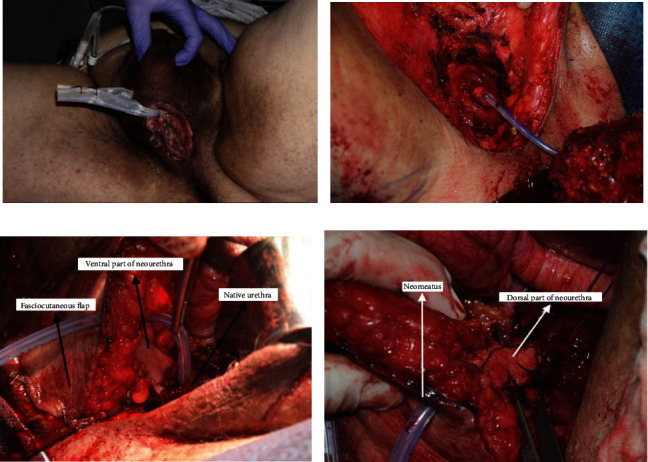
En-bloc excision of carcinoma and reconstruction of perineal defect. (a) Perineal wound indurated on palpation with drainage of purulent material. (b) En-bloc excision of tumor tissue. The prostatic apex can be seen. (c) Ventral side of neourethra. (d) Double buccal mucosa tubular graft. The neourethra is passed through the fasciocutaneous flap.

The surgical procedure consisted of different phases. Phase I: Total penectomy was performed, and en bloc removal of tumor tissue was done (Figures 2(a) and 2(b)). Phase II: Neourethra was performed with a double buccal mucosa graft, and internal pudendal fasciocutaneous flap was performed to cover the dead space and provide vascular support to the neourethra (Figures 2(c) and 2(d)).

The procedure lasted 9 hours and the estimated blood loss was 500 cc. During the postoperative period, the patient remained with the urinary catheter through the neourethra, a suprapubic catheter, and a rectal catheter to keep the receiving area of the flap as dry as possible. The patient was discharged on the twelfth day after surgery. During admission, he presented with a superficial dehiscence of the skin of the flap that was successfully treated with local paraffin dressings.

The patient presented a satisfactory postoperative period, being able to remove the urinary catheter and suprapubic catheter at 3 weeks prior urethrography, achieving spontaneous urination without involuntary leakage of urine.

The histological study reveals a moderately differentiated squamous cell carcinoma with keratinization and pleomorphic areas. The tumor shows neurotropism and frequent mitotic figures. The surgical edges were not compromised.

The patient received three cycles of adjuvant treatment with immunotherapy (cemiplimab 350 mg every 21 days), because was not subsidiary to treatment with platinum for renal failure. At 7 months postoperatively, he was diagnosed with pulmonary metastases associating massive pleural effusion that required thoracentesis in addition to pelvic and local lymph node disease. He passed out 9 months after surgery.

### 2.3. Case 3

A 78-year-old male patient with history of high blood pressure, dyslipidemia, and bulbar urethral stricture was treated by urethroplasty with dura graft in 1989. Subsequently, he underwent periodical dilations due to a recurrence of the stricture. The patient has perineal abscesses on multiple occasions that required surgical drainage. In the last drainage, a mamelon lesion suspected of carcinoma was observed adjacent to the perineal collection. The pathological result of the lesion reveals the existence of a well-differentiated squamous cell carcinoma. The extension study confirms the existence of a perineal collection that extends through both corpora cavernosa measuring 79 × 15 mm with solid and necrotic areas. In the first instance, the patient underwent a resection of the perineal tumor and a radical cystoprostatectomy. Subsequently, the perineal defect was covered with a fasciocutaneous flap. The pathological examination revealed the existence of a squamous cell carcinoma that affected both corpora cavernosa and corpus spongiosum, the perineal soft tissues, and the prostatic apex, leaving the surgical margins free of tumor. After one year of follow-up, the patient presented on imaging tests a recurrence in the pelvic bed that seemed to infiltrate the right ischiopubic branch and the right lateral wall of the rectum. The case is discussed in the multidisciplinary tumor committee, and the surgical treatment is decided. The patient underwent an abdominoperineal amputation, resection of the previous fasciocutaneous flap, and partial resection of the right ischiopubic branch. The perineal defect is covered with an anterolateral flap of the thigh.

The patient was discharged at 3 weeks without complications in the immediate postoperative period. After 2 years and 6 months of follow-up, the patient remains free of disease and with a complete functional recovery.

### 2.4. Case 4

A 78-year-old male patient with history of high blood pressure, a renal tumor that underwent partial nephrectomy, and a carcinoma in situ in the glans was treated by resurfacing. During the follow-up, the patient reported perineal and penile pain of months of evolution. The imaging tests performed revealed an abscess with a solid pole in the infrapubic region that affects the left corpus cavernosum. A core needle biopsy of the solid area was performed, diagnosing squamous cell carcinoma.

In the extension study, multiple indeterminate pulmonary nodules were identified. In the preoperative study, a significant growth of the perineal mass was observed, reaching 75 mm in its maximum diameter and affecting the left ischiopubic branch, adductor muscles, and ipsilateral gracilis. Pathological left inguinal lymph nodes were also observed.

Penile exploration and negative glans biopsy ruled out possible recurrence of penile carcinoma. The case was discussed in the multidisciplinary tumor committee, and the surgical treatment was decided.

The patient underwent total penectomy, resection of left isquiopubic branch, and partial resection of adductor muscles of the thigh and left inguinal lymph node dissection.

The perineal defect was covered with a contralateral gracilis flap.

The patient required postoperative wound debridement due to partial necrosis of the wound. The patient was discharged 3 weeks after the intervention with good functional recovery. The histological study revealed the existence of a moderately differentiated squamous cell carcinoma with a maximum diameter of 7.5 that affects the root of the penis and both corpora cavernosa. Inguinal lymph node dissection showed one metastatic node of the six resected. The patient received adjuvant chemotherapy treatment with cisplatin and 5-FU. At 6 months of follow-up, the patient presented a single left inguinal lymph node, subjected to excision with pathological results of metastasis of squamous cell carcinoma. After 17 months of follow-up, the patient presented pulmonary progression of the disease under treatment with the fourth line of chemotherapy treatment.

## 3. Discussion

We present a series of cases of 4 patients diagnosed with perineal squamous cell carcinoma undergoing surgical treatment. The uniqueness of this series of cases is supported by the lack of published case reports describing men patients with this malignancy treated surgically (Table 1). The two cases previously described were treated, one of them with chemoradiotherapy and the other with palliative treatment given the advanced state of the disease [1, 4]. From the published literature regarding squamous cell CUP in the pelvic region, the patients described were all women [1].

Carcinomas of unknown primary origin represent 3-5% of all malignancies and are defined as a heterogeneous group of tumors for which the primary origin cannot be established at the time of diagnosis. Within them, squamous carcinoma of unknown primary origin represents between 5 and 10% and is more frequent in the head and neck region, although they are also described at the uterine level and in iliac lymph nodes [1]. The presence of this type of tumors in the pelvic cavity is very rare, and the majority of cases described have occurred in women; there are only two cases reported in the literature of perineal carcinoma of unknown origin in men [1, 4].

Regarding the pathophysiology of these tumors, several articles have been written relating the inflammatory status with an increase in IL-1*β* that stimulates the NF-*κ*B pathway, highly involved in angiogenic and protumorigenic processes [5].

Patients with chronic infections of the perineal region, suppurative hydrosadenitis [6], urethral fistula, or any inflammatory/infectious process with a torpid evolution at this level should make us rule out the existence of an epidermoid carcinoma.

Transperineal biopsy proved to be a valid option for histological diagnosis. In experienced hands, the transperineal route allows to access to the small pelvis in complex situations [4]. Based on the limited literature data, it appears that squamous CUP outside of the head and neck region have a more favorable prognosis compared to other CUP and that these malignancies may respond well to a combination of surgical resection, when feasible, local radiotherapy, and platinum-based systemic chemotherapy [1].

Treatment regimens remain controversial, with limited cases to allow for consensus. Surgical intervention remains the leading form of therapy; however, approaches are case specific, depending on the location of the tumor and the degree of invasiveness [3].

In general, CUP has a poor prognosis, with a median survival time of less than six months and only about 15% of patients surviving after 1 year [1]. In our series, although with a very small number of cases, we have an overall survival of 75% with a median follow-up of 23 months which is much longer than those published to date.

## 4. Conclusion

Squamous cell CUP located in the male pelvis represent a rare entity, so the ideal diagnostic work-up, the optimal treatment strategies, and prognosis are not well determined. Radical surgical treatment, followed by adjuvant chemotherapy in patients with a localized tumor and with good baseline status, appears to be the best treatment regimen, achieving higher survival rates than those published to date.

## Figures and Tables

**Table 1:** Literature review.

Literature	Age	Presentation	Metastasis in other organs at diagnosis	Treatment	Follow up (months)
Chiec et al. 2014	52	Buttock pain	Liver, lung	Chemoradiation (cisplatin; 6000 centigray in 30 fractions)	12
Creta et al. 2017	78	Urethral stricture and perineal abscess	—	Palliative colostomy	3
Present	62	Urethral stricture and perineal abscess	Inguinal lymph nodes	Surgery + chemotherapy (carboplatin/gemcitabine)	36
Present	67	Urethral stricture and perineal abscess	—	Surgery + immunotherapy (cemiplimab 350 mg IV)	9
Present	78	Urethral stricture and perineal abscess	—	Surgery	30
Present	78	Penile pain, perineal abscess	Inguinal lymph nodes	Surgery + chemotherapy (cisplatin + 5-Fluoracil)	17

## Data Availability

The data used to support the findings of this study are included within the article.
